# CTCF Point Mutation at R567 Disrupts Mouse Heart Development via 3D Genome Rearrangement and Transcription Dysregulation

**DOI:** 10.1111/cpr.13783

**Published:** 2024-12-16

**Authors:** Huawei Ren, Hongxin Zhong, Jie Zhang, Yuli Lu, Gongcheng Hu, Weixun Duan, Ning Ma, Hongjie Yao

**Affiliations:** ^1^ College of Veterinary Medicine Shanxi Agricultural University Jinzhong China; ^2^ State Key Laboratory of Respiratory Disease Guangzhou Institutes of Biomedicine and Health, Chinese Academy of Sciences Guangzhou China; ^3^ State Key Laboratory of Respiratory Disease The First Affiliated Hospital of Guangzhou Medical University Guangzhou China; ^4^ School of Basic Medical Sciences Guangzhou Medical University Guangzhou China; ^5^ Department of Basic Research Guangzhou National Laboratory Guangzhou China; ^6^ Department of Cardiovascular Surgery Xijing Hospital Xi'an China

**Keywords:** 3D chromatin architecture, CTCF mutation, embryonic heart development, mouse, transcriptional regulation

## Abstract

CTCF plays a vital role in shaping chromatin structure and regulating gene expression. Clinical studies have associated CTCF mutations with congenital developmental abnormalities, including congenital cardiomyopathy. In this study, we investigated the impact of the homozygous CTCF‐R567W (*Ctcf* ^*R567W/R567W*^) mutation on cardiac tissue morphogenesis during mouse embryonic development. Our results reveal significant impairments in heart development, characterised by ventricular muscle trabecular hyperplasia and reduced ventricular cavity sizes. We also observe a marked downregulation of genes involved in sarcomere assembly, calcium ion transport, and mitochondrial function in heart tissues from homozygous mice. Furthermore, the *Ctcf* ^*R567W/R567W*^ mutation disrupts CTCF's interaction with chromatin, resulting in alterations to topologically associating domain (TAD) structure within specific genomic regions and diminishing crucial promoter‐enhancer interactions necessary for cardiac development. Additionally, we find that the heterozygous CTCF‐R567W (*Ctcf* ^*+/R567W*^) mutation significantly compromises cardiac contractility in 8‐week‐old mice. This study elucidates the mechanism by which the CTCF‐R567W mutation hampers cardiac development, underscoring the essential role of CTCF‐R567 in embryonic heart development and maturation.

## Graphical abstract

The *Ctcf^R567W/R567W^* mutation disrupted the TAD structure specific to cardiac genes and reduced the interaction between the promoter of cardiac‐specific gene and its enhancer, finally causing the cardiac morphological abnormalities during mouse embryonic development.

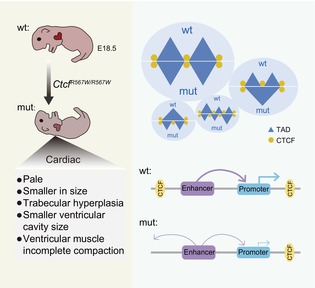

## 1 Introduction

Topologically associating domains (TADs) are fundamental structural units that organise chromatin, exhibiting preferential interactions and distinct boundaries, often adopting a ring‐like conformation [1, 2]. The CCCTC‐binding factor (CTCF) functions as a chromatin organiser, anchoring chromatin loops and establishing the boundaries [3, 4]. CTCF contributes to TAD formation by creating loops and coordinating the three‐dimensional chromatin structure [5, 6]. CTCF's biological function is mediated through its binding to specific DNA motifs, facilitated by its structure comprising 11 tandemly arranged zinc fingers (ZFs). ZFs 4–7 represent the core consensus, containing the M1/core (C) motif, while ZFs 9–11 encompass the M2/upstream (U) motif [7, 8]. Alterations to specific CTCF sites can disrupt normal long‐distance chromatin interactions, affecting enhancer‐promoter (EP) contact and influencing biological development and tumour gene expression [9, 10, 11, 12].

The association between deleterious variations in CTCF and clinical genetic mutations has garnered significant attention [13, 14]. CTCF functions in early embryonic development [15, 16] as well as in postnatal and adult life stages [17, 18]. Previous studies have identified various CTCF variants with heterozygous mutations in paediatric clinical cases [19]. Several amino acid residues have been reported as missense mutations, with Arg567Trp mutation observed in two patients [20, 21]. The R567 residue of CTCF, located within the 11th zinc finger (ZF) domain of the CTCF protein, is highly conserved and participates in specific DNA interactions [8]. Notably, *Ctcf* gene mutations in clinical patients are primarily associated with neurodevelopmental disorders [20, 22]. In addition to the typical features of autosomal dominant mental retardation 21 (MRD21), CTCF mutations also manifest as a broader spectrum of organ developmental abnormalities, affecting the teeth, fingers, bones, heart, urinary, and reproductive systems [10, 19, 22]. Two paediatric patients with *CTCF* mutations have been reported to exhibit cardiac abnormalities, including patent ductus arteriosus and mitral valve defect [21, 23]. The incidence of heart defects during human heart formation is 1%–2%, indicating that one to two babies out of every 100 are born with congenital heart disease [24]. The differentiation and maturation of cardiomyocytes, which are largely dependent on CTCF‐mediated genome interactions, underscore the significance of CTCF in cardiac development [25, 26, 27].

In our recent study [28], we found that CTCF Arg567Trp (CTCF‐R567W) homozygous mutant (*Ctcf* ^*R567W/R567W*^) mice exhibited lethality immediately after birth and significant developmental disorders in brain, heart, and lung tissues. Structurally, the R567W mutation induces a displacement of the ZF away from the phosphate backbone of DNA, disrupting the critical hydrogen bond between R567 and the DNA backbone [28]. The presence of the W567 residue may hinder the formation of an essential hydrogen bond between the neighbouring R566 residue and DNA bases, further compromising ZF11 binding to the U motif [28].

In this study, we aimed to elucidate the mechanism by which the *Ctcf* mutation affects mouse heart development. Our data indicate that *Ctcf* ^*R567W/R567W*^ homozygous mutations result in cardiac morphological defects and stunted development in the embryo, while adult CTCF‐R567W heterozygous (*Ctcf* ^*+/R567W*^) mice exhibit impaired cardiac contractility. At the molecular level, the *Ctcf* ^*R567W/R567W*^ mutation disrupts the TAD structure specific to cardiac genes and reduces the interaction between the promoter of the cardiac‐specific gene *Lmod2* and its enhancer, thereby altering the expression of genes associated with normal heart development.

## 2 Materials and Methods

### 2.1 Animals

C57BL/6N mice carrying the CTCF‐R567W mutation were generated via CRISPR/Cas9‐mediated gene editing by Cyagen Biosciences in Guangzhou, China [28]. The mice were housed in a specific pathogen‐free (SPF) facility, maintaining a constant temperature of 25 C ± 1°C and humidity of 50% ± 10% under a 12‐h light–dark cycle, with unrestricted access to sterilised water and food. All animal experiments were conducted in accordance with the National Institutes of Health Guidelines for the Care and Use of Laboratory Animals. The protocol was approved by the Committee on the Ethics of Animal Experiments at the Guangzhou Institutes of Biomedicine and Health, Chinese Academy of Sciences (IACUC number: 2023085).

### 2.2 Sample Harvesting

Pregnant mice were anaesthetised with a 1.2% intraperitoneal (*i.p*.) injection of Avertin (2,2,2‐tribromoethanol) (Cat#T48402, Sigma) and subsequently euthanised by cervical dislocation. The embryos were extracted, and their hearts were swiftly excised post‐decapitation and placed in cold PBS to remove excess blood. Following isolation, each heart tissue sample was collected in a single 1.5‐mL microcentrifuge tube and snap‐frozen in liquid nitrogen. Tissue samples were preserved at −80°C until further processing or fixed in 4% paraformaldehyde (Cat#P0099, Beyotime) for sectioning.

### 2.3 Section Preparation

For paraffin sectioning, heart tissues were fixed in 4% paraformaldehyde at 4°C for 24 h, followed by dehydration in a gradient of ethanol (70% to 100%), transparentised with xylene, infiltrated with molten paraffin, and further embedded into paraffin blocks. Paraffin‐embedded tissue sections finally were produced using a microtome (Leica, Germany). For frozen sectioning, fixed heart samples were embedded in optimal cutting temperature (OCT) compound and stored at −80°C until completely solidified. Then, thin slices were obtained using a cryostat microtome (Leica, Germany).

### 2.4 Echocardiography and EdU Staining

Echocardiography was performed using a high‐resolution ultrasound system M90 SCI (Mindray Animal, China) to examine the hearts of 8‐week‐old mice. For EdU staining, the BeyoClick EdU‐594 kit (Cat#C0078, Beyotime) was utilised according to the manufacturer's instruction. EdU solution was intraperitoneally injected into pregnant mice at a dosage of 50 mg/kg of body weight. Heart tissues from the embryonic mice were collected for frozen section preparation after 2 h. Tissue sections were permeabilised using a 0.3% Triton X‐100 solution at room temperature (RT) for 15 min, followed by incubation with Click reaction solution in the absence of light for 30 min. The tissues were subsequently labelled with Hoechst dye to visualise nuclei. Images were acquired using an Olympus cellSens Dimension Imaging System (IX73), and the cell proliferation rate was quantified as a percentage using ImageJ software.

### 2.5 HE Staining and Masson Staining

Paraffin sections (3–4 μm) were placed in an oven at 55°C for 2 h, followed by staining with either HE (Cat#G1005, Servicebio) or Masson's Trichrome (Cat#G1006, Servicebio) according to the manufacturer's instructions. The sections were first dewaxed in xylene and then hydrated in a gradient of ethanol from 100% to 75%. For HE staining, the nuclei were stained with haematoxylin, followed by dehydration in a gradient of ethanol from 70% to 100%, and subsequent staining with eosin. For Masson staining, the sections were sequentially stained with solutions A‐F, followed by dehydration in anhydrous ethanol and n‐butanol. Finally, the sections were transparentised in xylene and sealed with neutral resin, allowing them to air‐dry. Images were captured using a bright field microscope or a panoramic MIDI biopsy scanner (3D HISTECH).

### 2.6 Immunofluorescence Labelling and WGA Staining

Frozen heart sections were incubated with PBS containing 0.3% Triton X‐100 at RT for 15 min, followed by immunostaining with a blocking buffer (Cat#P0102, Beyotime) for 1 h to prevent nonspecific antibody binding. Primary antibodies were diluted in the blocking buffer and incubated with the samples for 2 h at RT. Heart samples were then incubated with Alexa Fluor‐conjugated secondary antibodies (Invitrogen), protected from light for 1 h at RT. Nuclei were visualised with DAPI for 10 min in the dark. For Wheat Germ Agglutinin (WGA, Cat#FL‐1021, Vector) staining, sections were incubated in WGA solution (50 μg/mL) for 30 min. Images were acquired using a confocal microscope (Carl Zeiss LSM 800, Germany).

### 2.7 TUNEL Assay

Cardiomyocyte apoptosis was detected using a TUNEL kit (Cat#C1086, Beyotime) following the manufacturer's protocol. Samples were fixed in 4% paraformaldehyde for 30 min and subsequently treated with 0.3% Triton X‐100 for 10 min. An appropriate volume of TUNEL detection solution was then added to the samples and incubated at 37°C for 60 min in the absence of light. Nuclei were counterstained with DAPI, and images were captured using an Olympus Microimaging System (IX73).

### 2.8 qRT‐PCR

Total RNA was extracted using TRIzol reagent (Cat#TR118, Molecular Research Center). One microgram of total RNA was reverse‐transcribed into cDNA using a cDNA synthesis kit (Cat#R323‐01, Vazyme). qRT‐PCR assays were performed using 2 × RealStar Power SYBR qPCR Mix (Cat#A311‐10, GenStar), with *Gapdh* serving as the reference gene. Relative gene expression was calculated using the 2^−(ΔΔCt)^ method. The primers used in this study are listed in Data S1.

### 2.9 Western Blot

To obtain protein extracts, cardiac tissue was homogenised in cold PBS using an electric grinder. The supernatant was collected by centrifugation at 12,000 rpm for 10 min. The tissue fragments were resuspended in RIPA buffer (0.1% SDS, 1% Triton X‐100, 150 mM KCl, 50 mM Tris–HCl [pH 7.4], 1 mM EDTA, 1 mM PMSF and 1× protease inhibitor cocktail) and lysed on ice for 30 min. The total soluble protein was obtained by centrifugation at 12,000 rpm at 4°C for 10 min. Subsequently, the entire supernatant was collected for protein immunoblot analysis.

After SDS‐PAGE, proteins were transferred onto a polyvinylidene difluoride (PVDF) membranes (Cat#ISEQ00010, Millipore). The membranes were blocked with 5% milk in TBST buffer and incubated with primary antibodies for 2 h at RT or overnight at 4°C. Following three washes in TBST buffer, membranes were incubated with secondary antibodies for 1 h at RT and washed three times with TBST buffer. Enhanced chemiluminescence (ECL) substrate solution (Cat#P10060, Ncm Biotech) was added for chemiluminescence detection, and its intensity was quantitated using a chemiluminescence imager (MiniChemi 910). The grey‐scale values of protein bands were measured using the ImageJ software.

### 2.10 Antibodies

The following antibodies were used in this study: Rabbit anti‐cTnT (1:200, Cat#ab45932, Abcam) for immunocytochemistry. Rabbit anti‐VDAC (1:1000, Cat#D73D12, Cell Signalling Technology), Mouse anti‐MTCO1 (1:1000, Cat#ab14705, Abcam), Rabbit anti‐ATP5A1 (1:10000, Cat#14676‐1‐AP, Proteintech) and Mouse anti‐β‐Actin (1:5000, Cat#A2228, Sigma) for western blot.

### 2.11 ATP Detection

ATP levels in the samples were quantified using an ATP test kit (Cat#S0027, Beyotime Biotechnology). Tissue samples were homogenised in a lysis solution at the specified proportions. Following this, the homogenate was subjected to centrifugation, and the supernatant was collected for subsequent experiments. The ATP test solution was prepared in accordance with the manufacturer's instructions and added to the respective test wells. The mixture was then incubated at RT for 3–5 min. An ATP standard solution was prepared by diluting it to the appropriate concentrations, and a standard curve was generated based on the provided guidelines. Subsequently, 20 μL of either the sample or standard solution was added to the test wells or tubes for rapid mixing. The relative light units (RLU) were measured using a luminometer, with readings taken at intervals of at least 2 s. The concentration of ATP in the sample was determined by referencing the standard curve.

### 2.12 RNA Sequencing (RNA‐Seq)

E18.5 mouse heart tissue was homogenised thoroughly using an electric tissue homogeniser. Total RNA was extracted using TRIzol (Cat#TR118, Molecular Research Center). RNA‐seq library was prepared following the manufacturer's specifications (Cat#NR605, Vazyme Biotech). mRNA was captured using mRNA capture beads prior to fragmentation. The RNA fragments were reverse transcribed and subjected to library amplification for subsequent Illumina sequencing (Annoroad Gene Technology Co. Ltd.).

### 2.13 RNA‐Seq Data Analysis

Raw RNA‐seq data underwent quality control using Trim Galore (v0.6.10) to clip adapter and trim low‐quality reads. The resulting high‐quality reads were aligned to the *Mus musculus* genome (mm10) using the Spliced Transcripts Alignment to a Reference (STAR) tool (v2.7.4a) [29]. Gene expression quantification was performed with RSEM (v1.2.28) [30], generating gene‐level count data. Differentially expressed genes were identified using the DESeq2 package (v1.42.0) [31], with criteria of an absolute fold change > 2 and an adjusted *p*‐value < 0.01. Heatmaps illustrating gene expression patterns were generated using the pheatmap package (v1.0.12). Additionally, Gene Ontology (GO) enrichment analysis was conducted with the clusterProfiler package (v4.10.2) [32]. Circular visualisation of expression data was achieved through the ggtree package (v3.10.0) [33].

### 2.14 ChIP‐Seq Data Analysis

For differential peak analysis, MAnorm (v1.1.4) [34] was utilised. Peak distributions were visualised using the deepTools suite (v3.5.4) [35]. Annotation of the identified ChIP‐seq peaks was performed using ChIPseeker (v1.38.0) [36]. Changes in orientation of CTCF motifs pre‐and post‐mutation were analysed using the FIMO tool from the MEME suite (v5.5.4) [37].

### 2.15 BL‐Hi‐C Data Analysis

Differential analysis of TAD boundaries and insulation scores was conducted using FANC compare (v0.9.25b1). Boundary scores were classified as increased if the difference was > 2 and decreased if < −2. The circlize package (v0.4.15) [38] was employed to visualise differential boundary scores, while TAD structures were illustrated using the plotgardener package (v1.8.1) [39].

### 2.16 4C Sequencing (4C‐Seq)

4C‐seq experiments were conducted as previously described [40]. Thoroughly homogenised heart tissue was cross‐linked with 2% formaldehyde and quenched with 0.2 M glycine. DNA was digested in situ with nuclease DpnII. 500 ng of DNA served as a template, which was linearly amplified using 5′ biotin‐labelled probes. The ssDNA was enriched using Dynabeads M‐280 magnetic beads. Illumina sequencing adaptors were introduced, followed by PCR amplification using target‐specific primers. Finally, 4C libraries were sequenced on the Illumina NovaSeq platform (Annoroad Gene Technology Co. Ltd.). All viewpoint‐specific primers for 4C‐seq are listed in DATA S1.

### 2.17 4C‐Seq Data Analysis

Adapters and low‐using TrimGalore. The trimmed reads were processed with cutadapt to select reads containing primer sequences at the 5′ end of read 1 [41], and the primer sequences were removed using the parameters “‐g primer –discard‐untrimmed”. Selected reads were mapped to the mouse mm10 genome using bowtie2 with the parameters “–very‐sensitive –end‐to‐end –no‐unal”. Samtools was used to generate bam files, filter out low‐quality mapped reads (MAPQ < 30), and remove PCR duplicates. The bam files were processed using the r3Cseq package to generate normalised bedGraph files [42], which were subsequently converted into bigWig files using the bedGraphToBigWig tool for visualisation.

### 2.18 Statistical Analysis

Statistical analyses were performed using GraphPad Prism 9 software. Data are presented as the averaged (mean) ± standard deviation (SD) with at least three independent replicates. Student's *t* test (unpaired, two‐sided) was used to analyse significant differences between two groups, while one‐way ANOVA was employed for comparisons among three groups. Differences were deemed significant at a *p*‐value of < 0.05. *p*‐values were indicated in each figure (*p < 0.05, **p < 0.01, ***p < 0.001 and ****p < 0.0001).

## 3 Results

### 3.1 Homozygous CTCF Mutation at R567W Leads to Cardiac Development Retardation in Embryonic Mice

**FIGURE 1:**
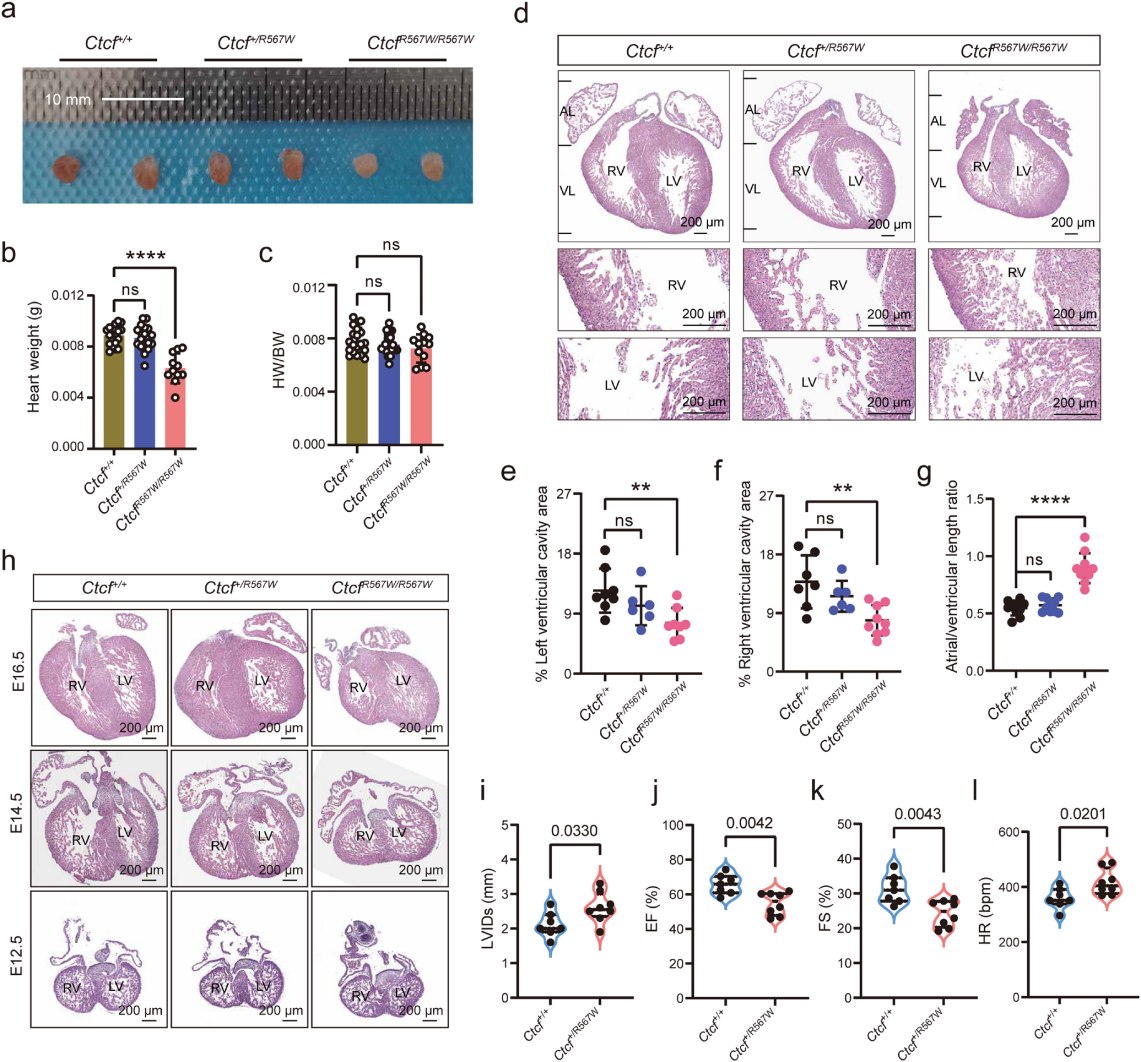
*Ctcf* ^*R567W/R567W*^ results in retardation of cardiac development in E18.5 mice. (a) Representative images of embryonic mouse hearts from wild‐type, *Ctcf* ^*+/R567W*^, and *Ctcf* ^*R567W/R567W*^ mice at E18.5. Scale bars, 10 mm. (b), Statistics of heart weight among wild‐type, *Ctcf* ^*+/R567W*^, and *Ctcf* ^*R567W/R567W*^ mice at E18.5 (*n* = 17 for *Ctcf* ^*+/+*^, *n* = 18 for *Ctcf* ^*+/R567W*^, and *n* = 11 for *Ctcf* ^*R567W/R567W*^). (c) Statistical analysis of the cardiac organ ratio in wild‐type, *Ctcf* ^*+/R567W*^, and *Ctcf* ^*R567W/R567W*^ mice at E18.5 (*n* = 20 for wild‐type, *n* = 20 for *Ctcf* ^*+/R567W*^, and *n* = 12 for *Ctcf* ^*R567W/R567W*^). HW, heart weight; BW, body weight. (d) Representative HE staining images of hearts from wild‐type, *Ctcf* ^*+/R567W*^, and *Ctcf* ^*R567W/R567W*^ mice at E18.5. Enlarged views of both the left and right ventricles are shown below. RV, right ventricle; LV, left ventricle; AL, atrial length; VL, ventricular length. Scale bars, 200 μm. (e–g) Quantification of cardiac phenotypes in wild‐type, *Ctcf* ^*+/R567W*^, and *Ctcf* ^*R567W/R567W*^ mice at E18.5. Shown are the percentage of left ventricular cavity area (e), percentage of right ventricular cavity area (f), and atrial/ventricular length ratio (g) with *n* = 6–10 for each genotype. (h) Representative HE staining images of hearts from wild‐type, *Ctcf* ^*+/R567W*^, and *Ctcf* ^*R567W/R567W*^ mice at E16.5, E14.5, and E12.5. RV, right ventricle; LV, left ventricle. Scale bars, 200 μm. (i–l), Echocardiographic analysis of wild‐type (*n* = 7) and *Ctcf* ^*+/R567W*^ mice (*n* = 8) at 8 weeks of age. Exact *p*‐values are indicated in the figure. LVIDs, left ventricular internal dimension at end‐systole (i); EF, ejection fraction (j); FS, fractional shortening (k); HR, heart rate (l). All data are presented as mean ± SD. One‐way ANOVA with Dunnett's multiple comparisons test (b, c, and e–g) and two‐tailed unpaired *t*‐tests (i–l); **p < 0.01, ****p < 0.0001, ns, not significant. Detailed statistical data are available in the Source data.

To investigate the biological impact of the CTCF‐R567W mutation on cardiac development, we examined the hearts of E18.5 embryos across three genotypes. While the hearts of wild‐type and heterozygous (*Ctcf* ^*+/R567W*^) mice showed no discernible abnormalities, those of *Ctcf* ^*R567W/R567W*^ mice appeared pale, smaller, and relatively lighter (Figure 1a,b). This observation indicates that the R567W mutation of CTCF severely impairs cardiac development in embryonic mice.

Although we observed a reduction in weight across other parenchymal organs, including the liver, kidney, spleen, lung, and brain in *Ctcf* ^*R567W/R567W*^ mutants at E18.5 (Figure S1a–e), haematoxylin and eosin (HE) staining revealed no obvious morphological differences in these organs (Figure S1f) [28]. Meanwhile, the heart coefficient ratio (heart weight/body weight, HW/BW) showed no significant difference (Figure 1c). Further, HE staining experiments unveiled abnormal cardiac morphology and pronounced ventricular muscle trabecular hyperplasia in CTCF^*R567W/R567W*^ mutant mice compared to both *Ctcf* ^*+/+*^ and *Ctcf* ^*+/R567W*^ mice (Figure 1d). Quantification of heart morphology discrepancies demonstrated a significant reduction in both left and right ventricular cavity areas in homozygotes, along with a notable increase in the atrial/ventricular length ratio (Figure 1e–g), indicating that CTCF^*R567W/R567W*^ led to decreased ventricular volume.

To explore other common cardiomyopathies in this mutant mouse model, we used wheat germ agglutinin (WGA) to label myocardial cell membranes. Homozygous mice displayed cardiomyocyte hypertrophy, while heterozygous cardiomyocytes exhibited a cross‐sectional area similar to wild‐type mice (Figure S1g,h). Masson staining revealed that neither *Ctcf* ^*R567W/R567W*^ nor *Ctcf* ^*+/R567W*^ mutations induced cardiac fibrosis in mice at E18.5 (Figure S1i), and no excessive fibrosis was observed in the hearts of 8‐week‐old adult wild‐type and heterozygous mice (Figure S1j,k).

We next sought to determine at which stage of embryonic development these abnormal cardiac manifestations appeared in the mutant mice. Our HE staining data showed that hearts with homozygous mutations exhibited significant ventricular dysplasia, whereas those with heterozygous mutations displayed morphology similar to wild‐type mice at the E16.5 and E14.5 stages, respectively (Figure 1h), consistent with the cardiac phenotype observed at E18.5. However, hearts with both heterozygous and homozygous mutations exhibited morphological characteristics indistinguishable from those in wild‐type mice at the E12.5 stage (Figure 1h), suggesting that *Ctcf* ^*R567W/R567W*^‐induced cardiac dysplasia manifests after E12.5. These findings indicate that *Ctcf* ^*R567W/R567W*^ results in stunted cardiac development in embryos.

### 3.2 CTCF Heterozygous Mutation at R567W Leads to Impaired Cardiac Contractile Function in Adult Mice

Although *Ctcf* ^*+/R567W*^ heterozygous mice displayed no overt morphological abnormalities and appeared to undergo normal development, we aimed to evaluate whether the CTCF‐R567W mutation affected heart function. Echocardiography was performed on both wild‐type and *Ctcf* ^*+/R567W*^ mutant mice at 8 weeks of age. We observed a significant increase in left ventricular internal dimension at end‐systole (LVIDs) in *Ctcf* ^*+/R567W*^ mice compared to wild‐type mice (Figure 1i). Additionally, our data revealed a notable decrease in ejection fraction (EF) and fractional shortening (FS), coupled with an elevated heart rate (HR) in *Ctcf* ^*+/R567W*^ mice (Figure 1j–l). These findings suggest that *Ctcf* ^*+/R567W*^ results in impaired ventricular systolic function relative to wild‐type mice.

Apart from these observed differences, interventricular septal thickness (IVS), left ventricular anterior wall thickness (LVAW), and left ventricular internal dimension at end‐diastole (LVIDd) showed no abnormalities in *Ctcf* ^*+/R567W*^ mice (Figure S1l–p), consistent with the phenotypes observed in heart sections of embryonic mice (Figure 1d,h). Collectively, our data indicate that *Ctcf* ^*+/R567W*^ compromises heart contractile function, while this heterozygous mutation does not visibly impact the morphological phenotype.

### 3.3 *Ctcf* ^*R567W*^ ^*/R567W*^ Significantly Downregulates the Expression of Genes Associated With Cardiac Morphogenesis and Myocardial Contraction

**FIGURE 2:**
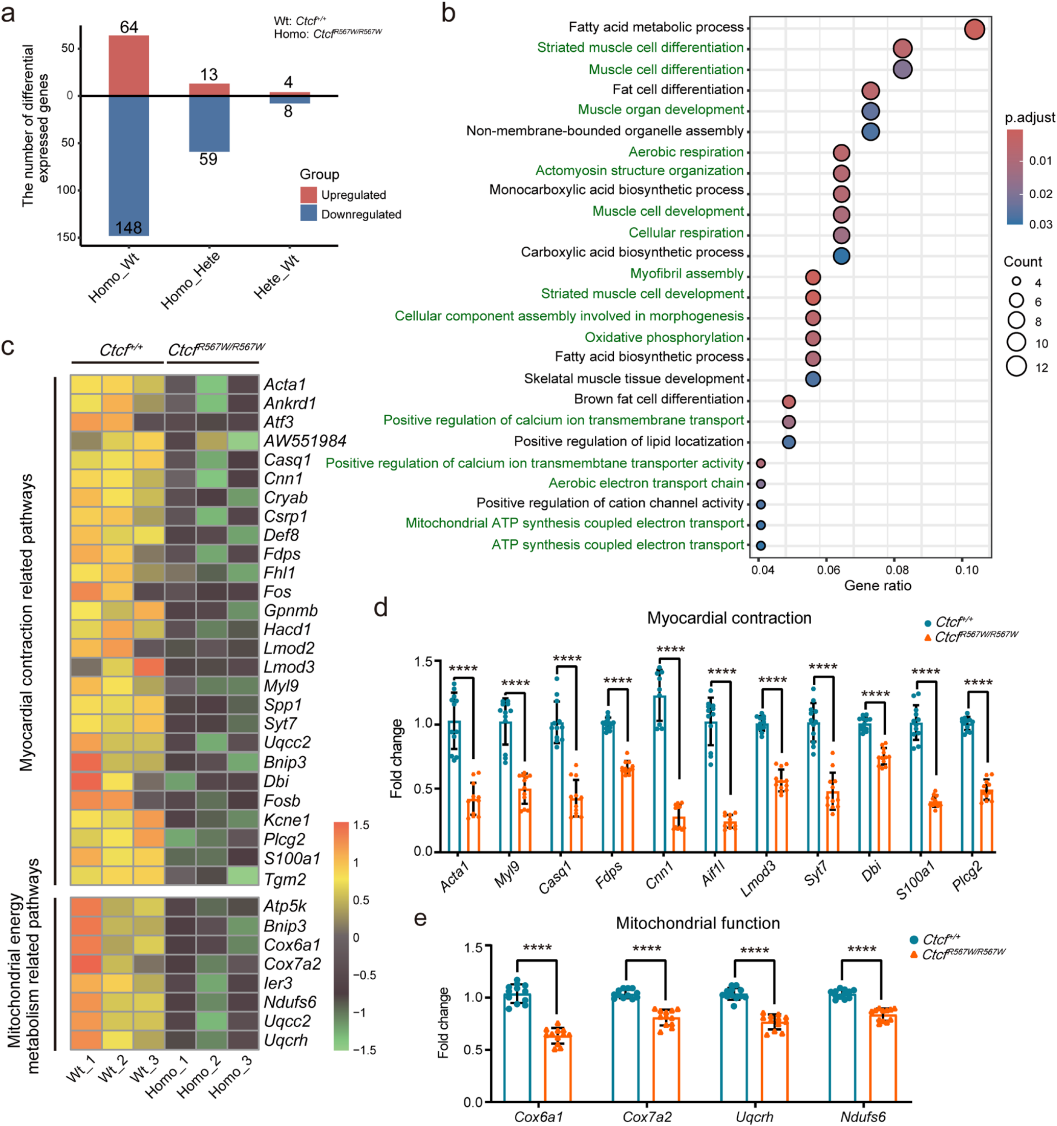
Impact of *Ctcf* ^*R567W/R567W*^ mutation on cardiac gene expression in E18.5 mice. (a), The number of differentially expressed genes (DEGs) among wild‐type, *Ctcf* ^*+/R567W*^, and *Ctcf* ^*R567W/R567W*^ mice in embryonic mouse hearts at E18.5. (b), Gene Ontology (GO) analysis of downregulated genes in wild‐type and *Ctcf* ^*R567W/R567W*^ mutant mice. Pathways related to myocardial contraction and mitochondrial energy metabolism are highlighted in green. (c), Heatmaps displaying enriched DEGs in embryonic hearts from wild‐type and *Ctcf* ^*R567W/R567W*^ mice at E18.5. Representative downregulated genes are listed in the box. (d‐e), RT‐qPCR analysis of mRNA levels of downregulated cardiac genes (*n* = 9–12 for *Ctcf* ^*+/+*^, *n* = 11–12 for *Ctcf* ^*R567W/R567W*^). All data are presented as mean ± SD. Multiple two‐tailed unpaired *t*‐tests (d and e). ****p < 0.0001. Detailed statistical data are available in the Source data.

To investigate whether the abnormal cardiac development induced by the *Ctcf* ^*R567W/R567W*^ mutation stemmed from alterations in proliferation or apoptosis in embryonic mice, we conducted EdU staining and TUNEL assay. Our findings revealed no significant impact of the *Ctcf* homozygous mutation on cellular proliferation or apoptosis of cardiomyocytes (Figure S2a–c). To elucidate the underlying mechanisms driving this mutation's effects on cardiac development, we performed RNA sequencing (RNA‐seq) experiments on hearts from E18.5 wild‐type, *Ctcf* ^*+/R567W*^, and *Ctcf* ^*R567W/R567W*^ mice. Comparison of the transcriptomes revealed minimal differences in differentially expressed genes (DEGs) between wild‐type and *Ctcf* ^*+/R567W*^ hearts (Figure 2a). Subsequently, we focused on the differences between homozygous and wild‐type mice to explore the mechanism of this mutation. *Ctcf* ^*R567W/R567W*^ hearts exhibited 64 upregulated genes and 148 downregulated genes (Figures 2a and S3a). Functional analysis of these DEGs revealed a significant enrichment of upregulated genes associated with kinase cascades and inflammatory immune responses in *Ctcf* ^*R567W/R567W*^ hearts (Figure S3b). However, downregulated genes were linked to striated muscle development, actin assembly, and cardiac morphogenesis (Figure 2b), including notable genes such as *Acta1*, *Myl9*, *Lmod2*, *Lmod3*, and *Cnn1* (Figure 2c). These findings correlate with the observed abnormal cardiac development phenotype in *Ctcf* ^*R567W/R567W*^ mutant mice. Furthermore, we noted a substantial decrease in genes associated with myocardial contractile function, including processes related to mitochondrial oxidative phosphorylation and calcium ion transmembrane signalling (Figure 2b,c).

To validate the transcriptional alterations induced by the CTCF‐R567W mutation, we performed reverse transcription followed by quantitative PCR (RT‐qPCR) analysis to assess the relative mRNA expression levels of genes in the cardiac tissues of E18.5 mice. Consistent with our expectations, the data revealed a significant reduction in the expression levels of genes associated with myocardial development and regulation of contraction function in *Ctcf* ^*R567W/R567W*^ mice compared to wild‐type counterparts (Figure 2d,e). Western blot experiments indicated no significant changes in the expression of mitochondrial outer membrane (OMM) protein VDAC, mitochondrial inner membrane (IMM) protein ATP5A, and mitochondrial matrix protein MTCO1 in *Ctcf* ^*R567W/R567W*^ hearts compared to wild‐type samples (Figure S3c,d), suggesting no effect on mitochondrial mass. However, the observed reduction in ATP production in cardiac tissue suggested potential mitochondrial dysfunction (Figure S3e), consistent with the GO analysis results described earlier (Figure 2b).

These findings collectively suggest that the homozygous CTCF‐R567W mutation disrupts normal gene expression in the heart of E18.5 mice.

### 3.4 Heterozygous CTCF‐R567W Mutation Leads to a Disorder of Mitochondrial Lipid Metabolism in the Adult Mouse Heart

**FIGURE 3:**
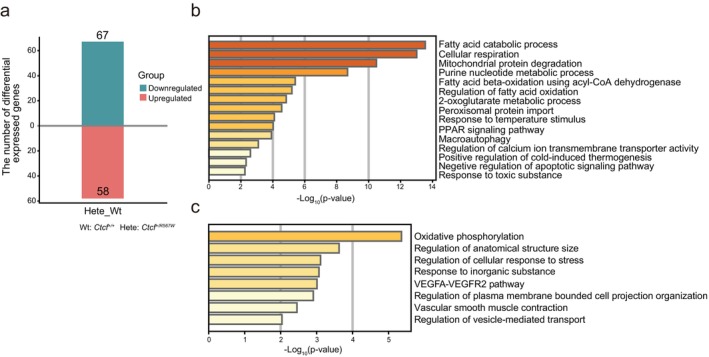
Effect of the *Ctcf* ^*+/R567W*^ mutation on myocardial contractile function in adult mice. (a), Number of DEGs identified between the hearts of 8‐week‐old wild‐type and *Ctcf* ^*+/R567W*^ mice. (b, c) GO analysis of downregulated (b) and upregulated (c) genes in the hearts of wild‐type and *Ctcf* ^*+/R567W*^ mutant mice at 8 weeks.

To investigate the underlying mechanism of impaired cardiac contractile function in adult heterozygous mice, we conducted RNA‐seq experiments on hearts from both 8‐week‐old wild‐type and heterozygous mutant mice. The results revealed that, compared with wild‐type mice, there were 67 genes exhibiting significant downregulation and 58 genes displaying significant upregulation in the hearts of heterozygous mice (Figure 3a). GO enrichment analysis identified that the majority of downregulated genes are associated with biological processes related to mitochondrial lipid metabolism (encompassing mitochondrial fatty acid β‐oxidation and the tricarboxylic acid cycle), respiratory electron transport, and calcium ion transmembrane transport (Figure 3b). Meanwhile, the upregulated genes are mainly enriched in the oxidative phosphorylation pathway, including *MTATP6*, *MTCOX3*, *MTND2*, *MTND4*, and *Pde4a*, which encode mitochondrial respiratory chain enzymes (Figure 3c). These findings suggest that the CTCF‐R567W mutation leads to disruption of mitochondrial lipid metabolism in the hearts of heterozygous mice during adulthood, ultimately resulting in weakened myocardial contractility.

### 3.5 *Ctcf* ^*R567W*^ ^*/R567W*^ Homozygous Mutation Disrupts CTCF Genomic Binding and Its‐Mediated Chromatin Boundary in Mouse Cardiac Tissue

**FIGURE 4:**
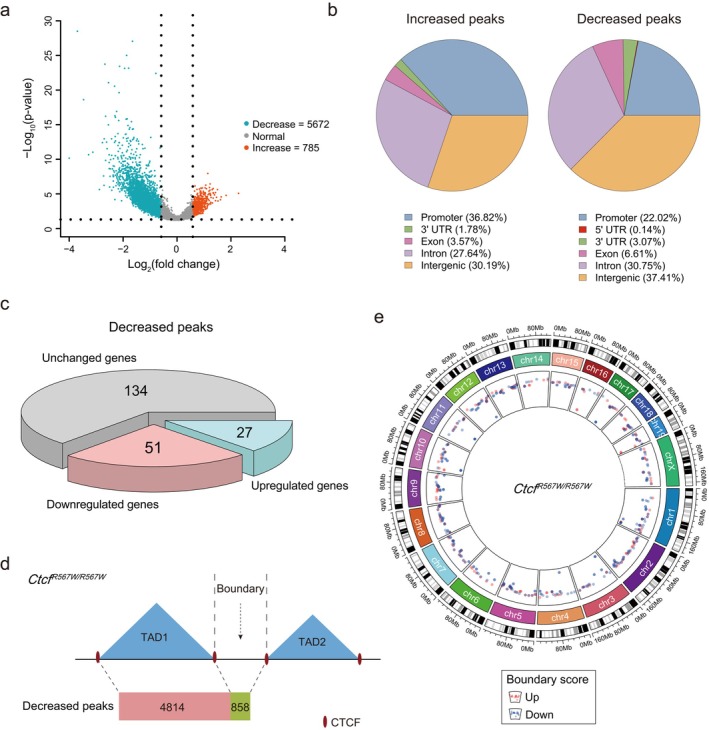
*Ctcf* homozygous mutation disrupts CTCF binding and its‐mediated boundary formation in mouse cardiac tissue. (a), Volcano plot illustrating the differential binding sites in wild‐type versus *Ctcf* ^*R567W/R567W*^ mutation. Orange and blue points signify regions with significantly increased or decreased binding sites, respectively. (b), Annotations of genomic locations of differential binding sites for CTCF peaks. Positions of promoters (+/− 2.5 kb around TSS), 3'UTR, 5'UTR, exons, introns, and intergenic regions are noted. (c) Pie chart depicting the number of DEGs with decreased CTCF peaks proximal to the gene. (d) Model diagram illustrating the distribution of CTCF peaks with decreased binding sites within TADs and at boundary regions. (e) Analysis of TAD boundary scores following *Ctcf* homozygous mutation. Each point represents a region, with red and blue points identifying regions with significantly increased or decreased TAD boundary scores, respectively (|scores| > 2).

Our recent investigation has noted a reduction in CTCF binding due to the CTCF‐R567W mutation, with minimal impact on compartments of E18.5 hearts compared to wild‐type [28]. To further elucidate the alterations in the CTCF binding profile in mouse heart induced by the CTCF‐R567W mutation, we conducted a detailed analysis. Comparing *Ctcf* ^*R567W/R567W*^ hearts to wild‐type hearts, we observed 785 increased CTCF peaks and 5672 decreased CTCF peaks (Figures 4a and S4a). Examination of the genomic distribution of these altered peaks revealed their predominant occurrence in intergenic, intron, and promoter regions (Figure 4b), suggesting a significant influence of the CTCF‐R567W mutation on the genomic binding of CTCF in mouse cardiac tissue.

To further elucidate the correlation between changes in CTCF peaks and alterations in gene expression due to the CTCF‐R567W mutation, we quantified the number of DEGs with decreased CTCF peaks proximal to the gene (Figure 4c). We observed 51 downregulated genes with decreased CTCF peaks nearby (Figures 4c and S4b), with approximately half of these genes being implicated in cardiac development or myocardial function (Figure S4b, in red). The tracks of the downregulated genes, *Ehd4* and *Uqcrh*, were presented as illustrative examples demonstrating a conspicuous decrease in CTCF peaks near the genes (Figure S4c). These results indicate a close correlation between the downregulation of myocardial‐related genes and the decrease in CTCF binding peaks.

CTCF is conventionally situated at chromatin region boundaries, contributing to the maintenance of TAD structure in mammals [10, 43]. We subsequently investigated the distribution of decreased CTCF peaks within chromatin TADs with or without the CTCF‐R567W homozygous mutation. Our analysis revealed that 4814 (84.9%) of the decreased CTCF sites were situated within the TAD region, while 858 (15.1%) were positioned at the TAD boundary regions in *Ctcf* ^*R567W/R567W*^ hearts (Figure 4d). Furthermore, we examined alterations in boundary scores across the entire genome following the CTCF‐R567W homozygous mutation. Our findings showed that 227 boundary scores increased while 346 decreased in genomic regions of *Ctcf* ^*R567W/R567W*^ hearts (Figure 4e). These results strongly indicate that the CTCF‐R567W homozygous mutation may indeed impact local chromatin boundaries.

### 3.6 CTCF‐R567W Homozygous Mutation Disturbs TAD Structure and Reduces the Interaction Between Gene Promoters and Enhancers in E18.5 Heart

**FIGURE 5:**
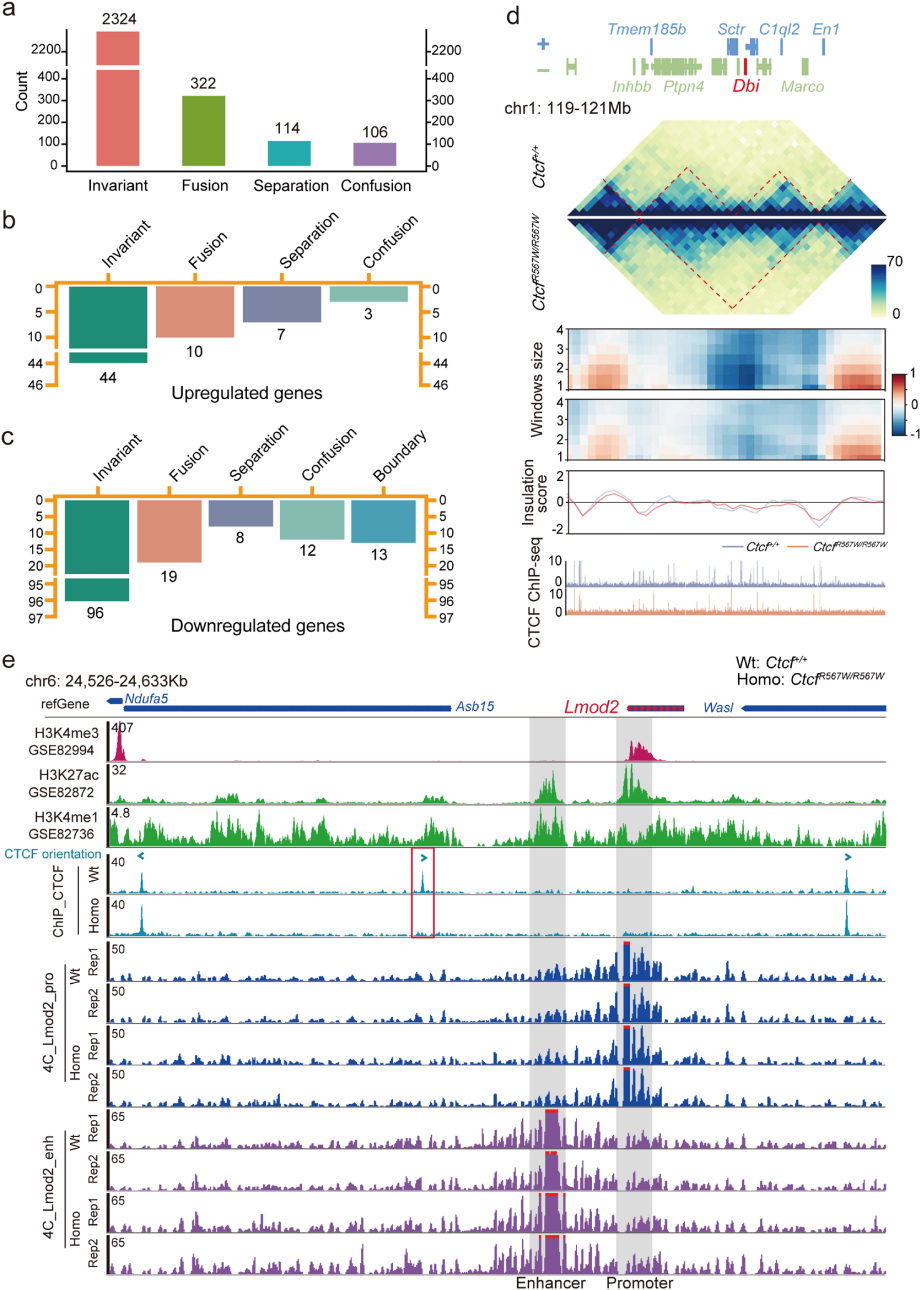
Impact of *Ctcf* homozygous mutation on TAD structure of cardiac‐specific gene *Dbi* and interaction between *Lmod2* gene promoter and its enhancer. (a) Number of TADs corresponding to the four types of TAD models induced by the homozygous mutation. (b, c) Statistical counts of the upregulated (b) and downregulated (c) genes in different type of TADs and boundary regions after the homozygous mutation. (d) Visualisation of the TAD structure, boundary scores, insulation scores, and CTCF binding within the vicinity of the *Dbi* gene in wild‐type versus *Ctcf* homozygous mutation. (e) 4C tracks illustrating interactions mediated by the *Lmod2* promoter and its potential enhancer in heart tissues of both wild‐type and *Ctcf* ^*R567W/R567W*^ mice. The red frame highlights the lost CTCF peak.

The failure of CTCF to bind at TAD boundaries disrupts their ability to segregate contacts between domains, leading to the merging of neighbouring TADs [4]. Upon our previous analysis, we have observed partial alterations in TAD structures due to the *Ctcf* ^*R567W/R567W*^ mutation [28]. Furthermore, we found that 322 TADs underwent fusion, 114 displayed separation, and 106 exhibited subTAD confusion, respectively (Figure 5a). Remarkably, 2324 TADs remained unchanged amidst these alterations in *Ctcf* ^*R567W/R567W*^ hearts (Figure 5a).

To explore the correlation between differentially expressed genes and changes in TAD structure, we quantified the alterations in TAD structure proximal to the differentially regulated genes (Figure 5b,c), particularly focusing on TAD structure adjacent to downregulated genes. Our statistical analysis revealed that 39 downregulated genes were associated with fusion, separation, and confusion induced by the *Ctcf* ^*R567W/R567W*^ mutation, while the TAD structures of 96 downregulated genes remained unchanged (Figure 5c). Notably, nearly half of these downregulated genes were linked to myocardial contraction and mitochondrial function, including *Cnn1*, *Myl9*, *Ndufs6*, *Atp5k*, *Lmod2*, *Dbi*, *Fdps*, *Syt7*, and *Cryab* among others (Figures S5, in red, and 2c).

To visually understand the relationship between DEGs, CTCF binding, and TAD structure, we illustrated with *Dbi* as an example. We examined the TAD structure, insulation fraction, and CTCF binding within the neighbouring 2 Mb regions (119–121 Mb). Our analysis revealed fusion of the TAD located in the *Dbi* locus with its adjacent TAD (Figure 5d). Specifically, the CTCF‐R567W mutation resulted in diminished CTCF binding near the *Dbi* gene, leading to reduced boundary insulation and subsequent fusion of two adjacent TADs (Figure 5d). These findings indicate that the homozygous CTCF‐R567W mutation induces abnormal expression of genes associated with myocardial structure and function by altering their associated TAD structures.

In the hearts of *Ctcf* ^*R567W/R567W*^ mice, we observed a decrease in the transcription level of the *Lmod2* gene (Figure 2c), along with the loss of CTCF binding at the upstream proximal CTCF peak (Figure 5e). CTCF facilitates EP interactions [44, 45]. To investigate the impact of CTCF mutation on the expression of heart‐associated genes, we utilised QHR‐4C (Quantitative high‐resolution chromosome conformation capture copy) to explore the potential interaction of the enhancer‐associated region of *Lmod2* (Figure 5e). Using the *Lmod2* promoter as a bait, we found that *Ctcf* ^*R567W/R567W*^ significantly attenuated the interaction between the potential upstream enhancer and the *Lmod2* promoter in mouse hearts (Figure 5e), consistent with the observed downregulation of *Lmod2* gene expression (Figure 2c). Furthermore, we examined alterations in the interactions of the potential enhancer with nearby elements, focusing on the enhancer as the viewpoint. Our findings indicate that the deletion of upstream CTCF sites in *Lmod2* leads to an obvious enhancement of the interaction between the potential enhancer and the upstream region in *Ctcf* ^*R567W/R567W*^ mouse hearts, compared to their wild‐type counterparts (Figure 5e). These results suggest that the CTCF‐R567W homozygous mutation alters the regulatory pattern of the potential enhancer of the *Lmod2* gene, resulting in a downregulation of gene expression.

Overall, our data demonstrate that the CTCF‐R567W homozygous mutation disrupts normal cardiac development by impairing CTCF binding and the interaction between enhancers and promoters of genes related to heart development, ultimately resulting in the downregulation of gene expression implicated in myocardial contraction and mitochondrial energy metabolism. In summary, R567 within CTCF plays a crucial role in embryonic heart development and maturation in mice.

## 4 Discussion

CTCF mutations inflict extensive impairments on various vital organs, including the brain, heart, skeletal muscles, bones, and genitourinary system [22]. In this study, histochemical experiments and multi‐omics analysis were conducted on mouse hearts harbouring the CTCF‐R567W point mutation. Our findings revealed that the CTCF‐R567W homozygous mutation significantly reduced CTCF's binding to the heart genome, leading to altered chromatin interactions. This dysregulation affected numerous key transcription factors involved in heart development, ultimately resulting in cardiac developmental arrest after E12.5 during the embryonic stage, accompanied by notable morphological defects.

Statistical analysis of organ and body weights in E18.5 mice demonstrated that CTCF‐R567W homozygous mutant mice generally exhibit smaller body and organ sizes, including the heart, compared to their wild‐type and heterozygous counterparts. Therefore, it is not surprising that there is no significant difference in the heart coefficient ratio. In heart sections of *Ctcf* ^*R567W/R567W*^ mice, a significantly smaller ventricular proportion and trabecular hyperplasia were observed, leading to narrowing of the ventricular cavity and notable birth defects such as ventricular muscle incomplete compaction, consistent with symptoms of left ventricular noncompaction cardiomyopathy (LVNC) [46, 47, 48]. Notably, patients with LVNC often present with other cardiomyopathies, such as hypertrophic cardiomyopathy (HCM), dilated cardiomyopathy (DCM), or heart failure (HF) [46, 49]. The CTCF‐R567W homozygous mice displayed symptoms of cardiomyocyte hypertrophy, corroborating previous reports [46, 49]. The stagnation of endocardial myocardium morphogenesis, attributed to the failure of the myocardial compaction pathway, resulted in postnatal incomplete cardiac myocyte densification [46, 49, 50]. Additionally, we observed a pallid and anaemic appearance in the hearts and entire bodies of *Ctcf* ^*R567W/R567W*^ mice. This observation suggests that the mutation may significantly impede cardiac haemodynamics and systemic blood circulation, resulting in inadequate myocardial perfusion and impairing various physiological functions [51, 52, 53].

Our transcriptomic analysis unveiled significant downregulation of genes involved in cardiomyocyte development and myofibril assembly in *Ctcf* ^*R567W/R567W*^ mice at E18.5. Genes responsible for ventricular noncompaction typically encode sarcomere or myocardial skeleton proteins [48, 54], potentially contributing to the observed myocardial compaction failure in *Ctcf* ^*R567W/R567W*^ hearts. Moreover, substantial downregulation of genes involved in calcium ion transport and processes related to mitochondrial energy metabolism, including fatty acid metabolism. The mitochondrial oxidative phosphorylation system plays a pivotal role in cellular metabolism, serving as the primary mechanism for energy generation in eukaryotic cells [55]. ATP, driven by the electron transport chain, is the principal energy carrier in nearly all cellular processes and the primary energy source for cardiomyocyte contraction [55]. Given the substantial energy demand for the heart's contraction and relaxation, the heart maintains energy reserves sufficient for only a limited number of beats [56]. Previous research has associated mitochondrial disorders with the progressive deterioration of myocardial structure and function, involving various theories, including mitochondrial DNA (mtDNA) mutation/damage, lipid metabolism disorders, mitochondrial calcium ion overload, and mitochondrial dynamic imbalance [57]. While our study did not uncover evidence that the CTCF‐R567W homozygous mutation directly affects mitochondrial structure, the significant downregulation of related genes strongly suggests a disruption in mitochondrial function.

In this study, while the heterozygous mutant hearts exhibited normal morphology, echocardiography data revealed a notable reduction in cardiac function characterised by impaired left ventricular muscle contraction and a significantly higher heart rate in adult *Ctcf* ^*+/R567W*^ mice compared to wild‐type counterparts. Transcriptome sequencing analysis of adult mice uncovered a significant downregulation of numerous genes associated with mitochondrial lipid metabolism in heterozygous mouse hearts, which is consistent with the observed impaired cardiac function. The mitochondrial electron transport chain employs oxidative phosphorylation via a series of electron transfer reactions to generate cellular ATP [58]. The rate of oxidative phosphorylation is significantly influenced by the ATP/ADP ratio. In the presence of mitochondrial dysfunction and reduced ATP levels, there is an upregulation in the rate of oxidative phosphorylation to meet cellular energy demands. In adult heterozygous mouse hearts, we observed a concomitant upregulation in the gene expression encoding mitochondrial respiratory chain enzymes, potentially serving as a compensatory mechanism aimed at enhancing oxidative phosphorylation. *Ctcf* ^*R567W/R567W*^ mice with evident morphological defects may exhibit more severe cardiac dysfunction. It is plausible that the significant cardiac dysfunction may contribute to the absence of homozygous *Ctcf* patients in clinical settings. Notably, our findings offer a potential explanation for the fatigue observed in adult *Ctcf* ^*+/R567W*^ mice during the rotating rod behavioural test [28].

Homozygous mutations in CTCF‐R567W in the embryonic heart can result in gene dysregulation and severe cardiac defects. However, this mutation does not induce extensive changes in TADs. Instead, the alterations appear to be localised, with approximately 81% of TADs remaining stable. Consistent with related studies by other researchers, *Ctcf* deletion led to severe fetal death and abnormal cardiac development without causing dysregulation of gene expression across a wide range of chromatin domains [25, 59]. The CTCF‐R567W homozygous mutation leads to the downregulation of crucial genes in the heart, although most of them show no changes in TAD structure, while their EP interactions are affected. In other words, CTCF facilitates tissue‐specific EP interactions of major regulators involved in cardiac development programs [25].

Lmod2 plays a pivotal role in regulating actin filament assembly and serves as a vital component of actin myofilaments in cardiac cells [60]. Our findings indicate that the loss of CTCF at the upstream region of the *Lmod2* gene results in enhanced interactions between nearby potential enhancer and upstream distal elements, while the regulation of the target gene (*Lmod2*) is relatively weakened. We speculate that this is a consequence of the loss of CTCF insulator function, leading to selective regulation of the enhancer.

In conclusion, this study revealed the molecular mechanism underlying heart abnormalities in CTCF‐R567W mice, which is of great significance for understanding the pathogenesis of heart disease and for future diagnosis and treatment strategies. Furthermore, our findings underscore the significance of CTCF‐R567 for normal development and maturation of the embryonic heart, providing additional insights into the guiding role played by CTCF‐mediated loops in regulating and maintaining cardiac gene expression.

## 5 Limitations of This Study

We acknowledge several limitations in our study. While echocardiography and RNA‐seq analysis demonstrated a significant impairment in cardiac function of heterozygous mice at 8 weeks of age compared to wild‐type mice, our findings revealed no significant differences in the transcription levels of genes in their hearts at E18.5. This phenomenon may be attributed to several factors. One possible explanation is the cumulatively biological effects, where subtle alterations at an early developmental stage (E18.5) gradually accumulate over time. Combined with the continuous high‐intensity workload of the heart, these cumulative effects could ultimately manifest as detectable cardiac dysfunction in adult mice. Additionally, we must consider the potential dosage effect of CTCF, a crucial structural protein for cell survival. Despite a substantial knockdown of CTCF levels (over 80%), the majority of chromatin structures appear to remain intact, leading to a comparatively modest impact on gene expression [61]. During the early developmental stages, compensatory mechanisms may effectively mask the initial effects of CTCF mutation. However, as the mice age and these compensatory mechanisms become inadequate, the underlying cardiac damage may become more evident. These limitations underscore the complexity of the relationship between genetic alterations and functional outcomes in cardiac development, necessitating further investigation to elucidate the mechanisms at play.

## Author Contributions

H.Y. conceived the project and designed the experiments. H.R., J.Z. and Y.L. performed the experiments. H.Z. and G.H. performed bioinformatic analysis. N.M. and W.D. contributed to the work. H.Y., H.R., J.Z. and H.Z. wrote the manuscript. H.Y. supervised this study.

## Conflicts of Interest

The authors declare no conflicts of interest.

## Supporting information


**Figure S1.** Effect of CTCF‐R567W mutation on cardiac phenotype and function in mice. (a–e), Organ weight statistics, including liver (a), brain (b), lung (c), kidney (d), and spleen (e) in wild‐type, *Ctcf* ^*+/R567W*^, and *Ctcf* ^*R567W/R567W*^ mice at E18.5 (*n* = 6–8 for *Ctcf* ^*+/+*^ and *Ctcf* ^*+/R567W*^; *n* = 3–5 for *Ctcf* ^*R567W/R567W*^). Exact *p*‐values are indicated in the figure. (f), Representative HE staining images of embryonic mouse liver, kidney, and spleen from wild‐type, *Ctcf* ^*+/R567W*^, and *Ctcf* ^*R567W/R567W*^ mice at E18.5. Scale bars, 100 μm (liver), 50 μm (kidney), and 60 μm (spleen). (g), Representative fluorescence images of immunostained cardiomyocytes with wheat germ agglutinin (WGA, green) in wild‐type, *Ctcf* ^*+/R567W*^, and *Ctcf* ^*R567W/R567W*^ mice at E18.5. Scale bars, 10 μm. (h) Quantitative analysis of g. At least 60 myocardial cells from 3 hearts were analysed for each genotype. (i), Representative Masson staining images in E18.5 hearts from wild‐type, *Ctcf* ^*+/R567W*^, and *Ctcf* ^*R567W/R567W*^ mice. Scale bars, 100 μm. (j,k), Representative images (j) and quantitative analysis (k) of Masson staining in the hearts of 8‐week‐old wild‐type and *Ctcf* ^*+/R567W*^ mice (*n* = 3 for each genotype). Scale bars, 100 μm. (l–p), Echocardiographic analysis of 8‐week‐old wild‐type (*n* = 7) and *Ctcf* ^*+/R567W*^ mice (*n* = 8). LVIDd, left ventricular internal dimension at end‐diastole (l); IVSd, interventricular septal thickness at end‐diastole (m); LVAWd, left ventricular anterior wall thickness at end‐diastole (n); IVSs, interventricular septal thickness at end‐diastole (o); LVAWs, left ventricular internal dimension at end‐systole (p). All data are presented as mean ± SD. One‐way ANOVA with Dunnett’s multiple comparisons test (a–e and h) and two‐tailed unpaired t‐tests (k–p); ****p < 0.0001, ns, not significant. Detailed statistical data are available in the Source data.

**Figure S2.** Effect of *Ctcf* ^*R567W/R567W*^ mutation on cardiomyocyte proliferation and apoptosis in E18.5 mice. (a, b) Schematic (top), representative images (left), and quantitative analysis (right) for EdU‐labelled proliferative cells (red) and cTnT‐stained cardiomyocytes (green) in E18.5 embryonic hearts from wild‐type, *Ctcf* ^*+/R567W*^, and *Ctcf* ^*R567W/R567W*^ mice. 16 enlarged fields of view were randomly selected for each genotype. Scale bars, 50 μm. (c) Representative images for TUNEL staining in E18.5 embryonic heart tissues from wild‐type, *Ctcf* ^*+/R567W*^, and *Ctcf* ^*R567W/R567W*^ mice. Scale bars, 50 μm. All data are presented as mean ± SD. Two‐tailed unpaired *t*‐tests (b); ns, not significant. Detailed statistical data are available in the Source data.

**Figure S3.** Effect of *Ctcf* homozygous mutation on cardiac gene expression in E18.5 mice. (a) Heatmap illustrating expression changes of genes in hearts from wild‐type and *Ctcf* ^*R567W/R567W*^ mice. (b) GO analysis of upregulated genes in wild‐type versus *Ctcf* ^*R567W/R567W*^ mice. (c–d), Western blot analysis (c) and quantification (d) of mitochondrial OMM proteins (VDAC), IMM proteins (ATP5A), and mtDNA‐encoded IMM proteins (MTCO1) in E18.5 embryonic hearts from wild‐type and *Ctcf* ^*R567W/R567W*^ mice (*n* = 3 for each genotype). Exact *p*‐values are indicated in the figure. (e) Measurement of ATP concentration in E18.5 embryonic hearts from wild‐type and *Ctcf* ^*R567W/R567W*^ mice (*n* = 10 for each genotype). Exact *p*‐values are indicated in the figure. All data are presented as mean ± SD. Two‐tailed unpaired *t*‐tests (d, e). Detailed statistical data are available in the Source data.

**Figure S4.** Correlation analysis between changes in CTCF peaks and alterations in gene expression in the CTCF‐R567W mutation. (a) Heatmaps displaying CTCF ChIP‐seq signals at regions exhibiting differential binding sites in wild‐type versus *Ctcf* ^*R567W/R567W*^ mutation. Average profiles are shown above the corresponding heatmaps. (b) Heatmaps revealing 51 downregulated genes with decreased CTCF peaks nearby. Genes related to myocardial development are highlighted in red. (c), ChIP‐seq signal tracks providing insights into differential CTCF occupancy near *Ehd4* and *Uqcrh* genes in wild‐type and *Ctcf* ^*R567W/R567W*^ mutation.

**Figure S5.** Association analysis of RNA‐seq and Hi‐C data. Ring heatmap illustrating downregulated genes adjacent to altered TADs. Genes involved in myocardial development pathways are highlighted in red.

**Data S1.** Lists of primer sequences used for RT‐qPCR and 4C experiments.

**Data S2.** DEGs from heart tissues RNA‐seq data.

**Data S3.** GO pathway analysis.

**Data S4.** MAnorm results.

**Data S5.** Source data.

## Data Availability

The RNA‐seq and 4C‐seq data reported in this article have been deposited in the Gene Expression Omnibus (GEO) database (GSE262508, GSE276744, and GSE276654), and in the Genome Sequence Archive database in the National Genomics Data Center (GSA) under accession code CRA015551. Published ChIP‐seq and BL‐Hi‐C data (GSE214692) were used in this paper. All the other data generated or analyzed during this study are included within the article and supporting information.
