# Supplementary figures and images for: CTCF Point Mutation at R567 Disrupts Mouse Heart Development via 3D Genome Rearrangement and Transcription Dysregulation
Source: Cell Prolif. 2024 Dec 16;58(4):e13783. doi: 10.1111/cpr.13783 (PMC11969252; doi:10.1111/cpr.13783)

Figure S1

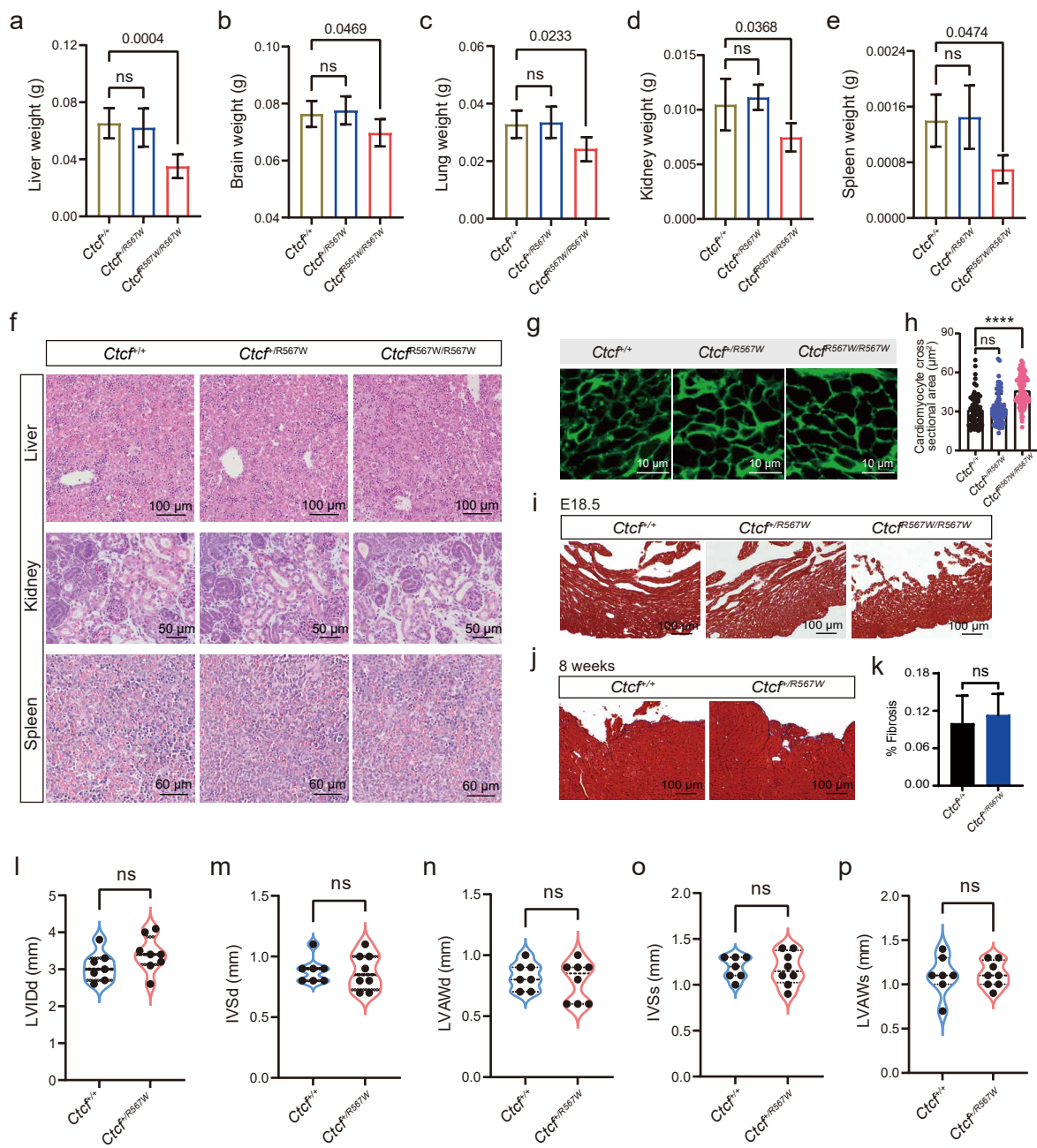

Supplement: Supplementary file 1 — Figure S1. Effect of CTCF‐R567W mutation on cardiac phenotype and function in mice. (a–e), Organ weight statistics, including liver (a), brain (b), lung (c), kidney (d), and spleen (e) in wild‐type, Ctcf +/R567W , and Ctcf R567W/R567W mice at E18.5 (n = 6–8 for Ctcf +/+ and Ctcf +/R567W ; n = 3–5 for Ctcf R567W/R567W ). Exact p‐values are indicated in the figure. (f), Representative HE staining images of embryonic mouse liver, kidney, and spleen from wild‐type, Ctcf +/R567W , and Ctcf R567W/R567W mice at E18.5. Scale bars, 100 μm (liver), 50 μm (kidney), and 60 μm (spleen). (g), Representative fluorescence images of immunostained cardiomyocytes with wheat germ agglutinin (WGA, green) in wild‐type, Ctcf +/R567W , and Ctcf R567W/R567W mice at E18.5. Scale bars, 10 μm. (h) Quantitative analysis of g. At least 60 myocardial cells from 3 hearts were analysed for each genotype. (i), Representative Masson staining images in E18.5 hearts from wild‐type, Ctcf +/R567W , and Ctcf R567W/R567W mice. Scale bars, 100 μm. (j,k), Representative images (j) and quantitative analysis (k) of Masson staining in the hearts of 8‐week‐old wild‐type and Ctcf +/R567W mice (n = 3 for each genotype). Scale bars, 100 μm. (l–p), Echocardiographic analysis of 8‐week‐old wild‐type (n = 7) and Ctcf +/R567W mice (n = 8). LVIDd, left ventricular internal dimension at end‐diastole (l); IVSd, interventricular septal thickness at end‐diastole (m); LVAWd, left ventricular anterior wall thickness at end‐diastole (n); IVSs, interventricular septal thickness at end‐diastole (o); LVAWs, left ventricular internal dimension at end‐systole (p). All data are presented as mean ± SD. One‐way ANOVA with Dunnett’s multiple comparisons test (a–e and h) and two‐tailed unpaired t‐tests (k–p); ****p < 0.0001, ns, not significant. Detailed statistical data are available in the Source data. [file CPR-58-e13783-s005.pdf]

Figure S2

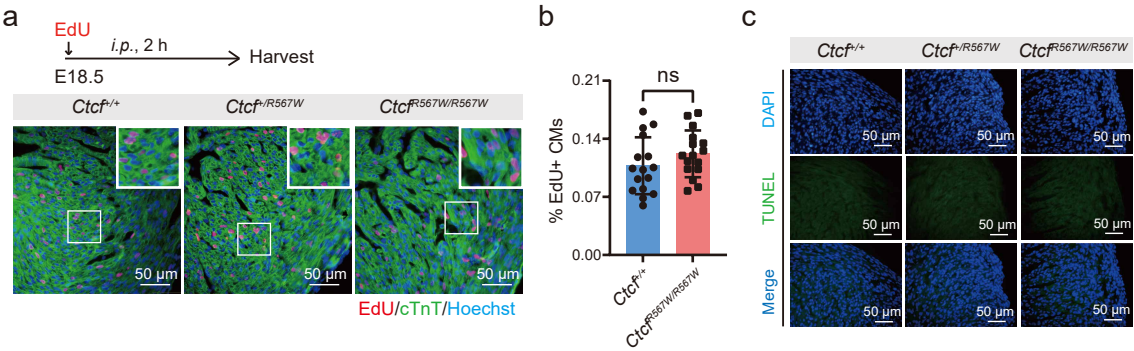

Supplement: Supplementary file 2 — Figure S2. Effect of Ctcf R567W/R567W mutation on cardiomyocyte proliferation and apoptosis in E18.5 mice. (a, b) Schematic (top), representative images (left), and quantitative analysis (right) for EdU‐labelled proliferative cells (red) and cTnT‐stained cardiomyocytes (green) in E18.5 embryonic hearts from wild‐type, Ctcf +/R567W , and Ctcf R567W/R567W mice. 16 enlarged fields of view were randomly selected for each genotype. Scale bars, 50 μm. (c) Representative images for TUNEL staining in E18.5 embryonic heart tissues from wild‐type, Ctcf +/R567W , and Ctcf R567W/R567W mice. Scale bars, 50 μm. All data are presented as mean ± SD. Two‐tailed unpaired t‐tests (b); ns, not significant. Detailed statistical data are available in the Source data. [file CPR-58-e13783-s007.pdf]

Figure S3

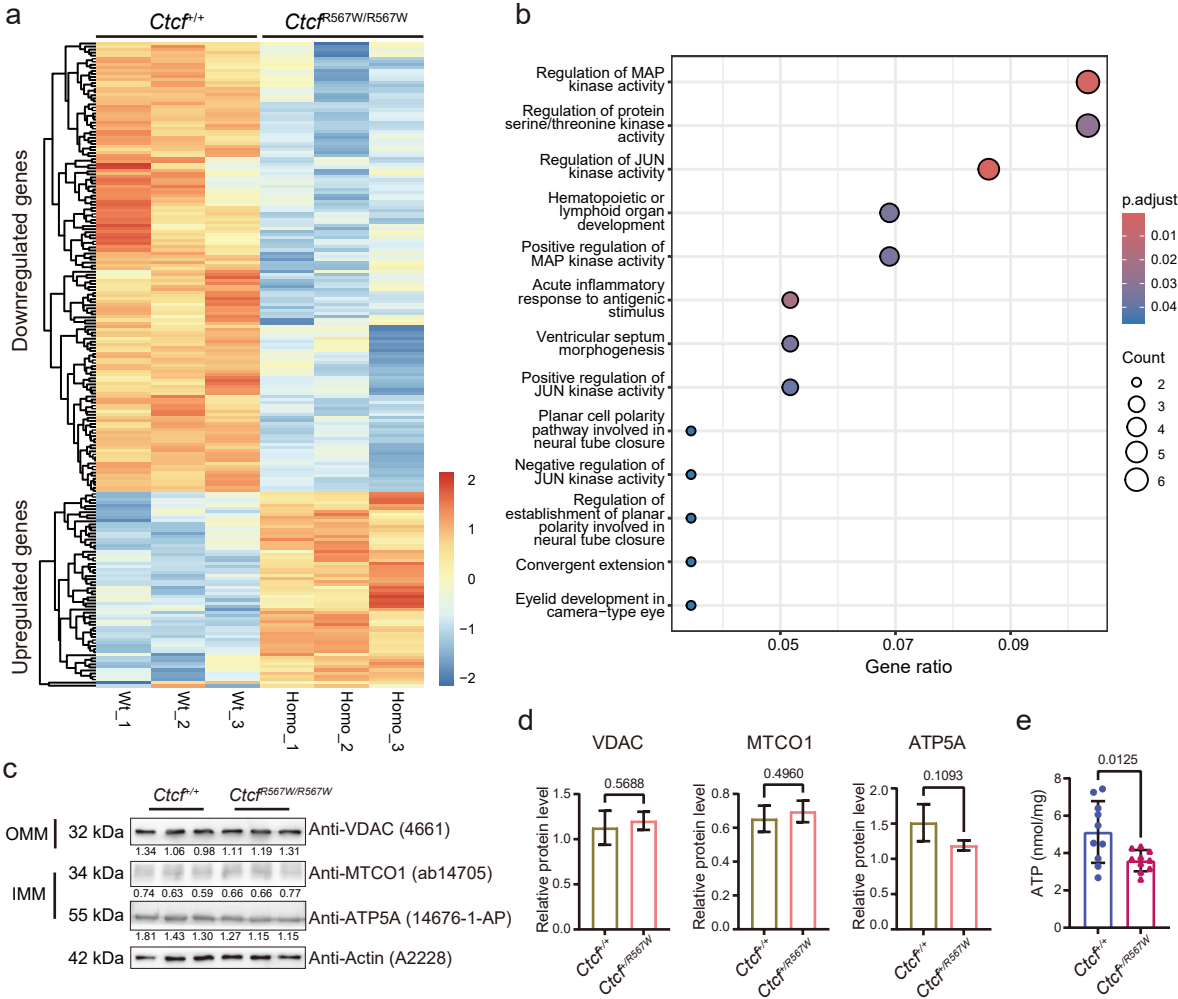

Supplement: Supplementary file 3 — Figure S3. Effect of Ctcf homozygous mutation on cardiac gene expression in E18.5 mice. (a) Heatmap illustrating expression changes of genes in hearts from wild‐type and Ctcf R567W/R567W mice. (b) GO analysis of upregulated genes in wild‐type versus Ctcf R567W/R567W mice. (c–d), Western blot analysis (c) and quantification (d) of mitochondrial OMM proteins (VDAC), IMM proteins (ATP5A), and mtDNA‐encoded IMM proteins (MTCO1) in E18.5 embryonic hearts from wild‐type and Ctcf R567W/R567W mice (n = 3 for each genotype). Exact p‐values are indicated in the figure. (e) Measurement of ATP concentration in E18.5 embryonic hearts from wild‐type and Ctcf R567W/R567W mice (n = 10 for each genotype). Exact p‐values are indicated in the figure. All data are presented as mean ± SD. Two‐tailed unpaired t‐tests (d, e). Detailed statistical data are available in the Source data. [file CPR-58-e13783-s003.pdf]

Figure S4

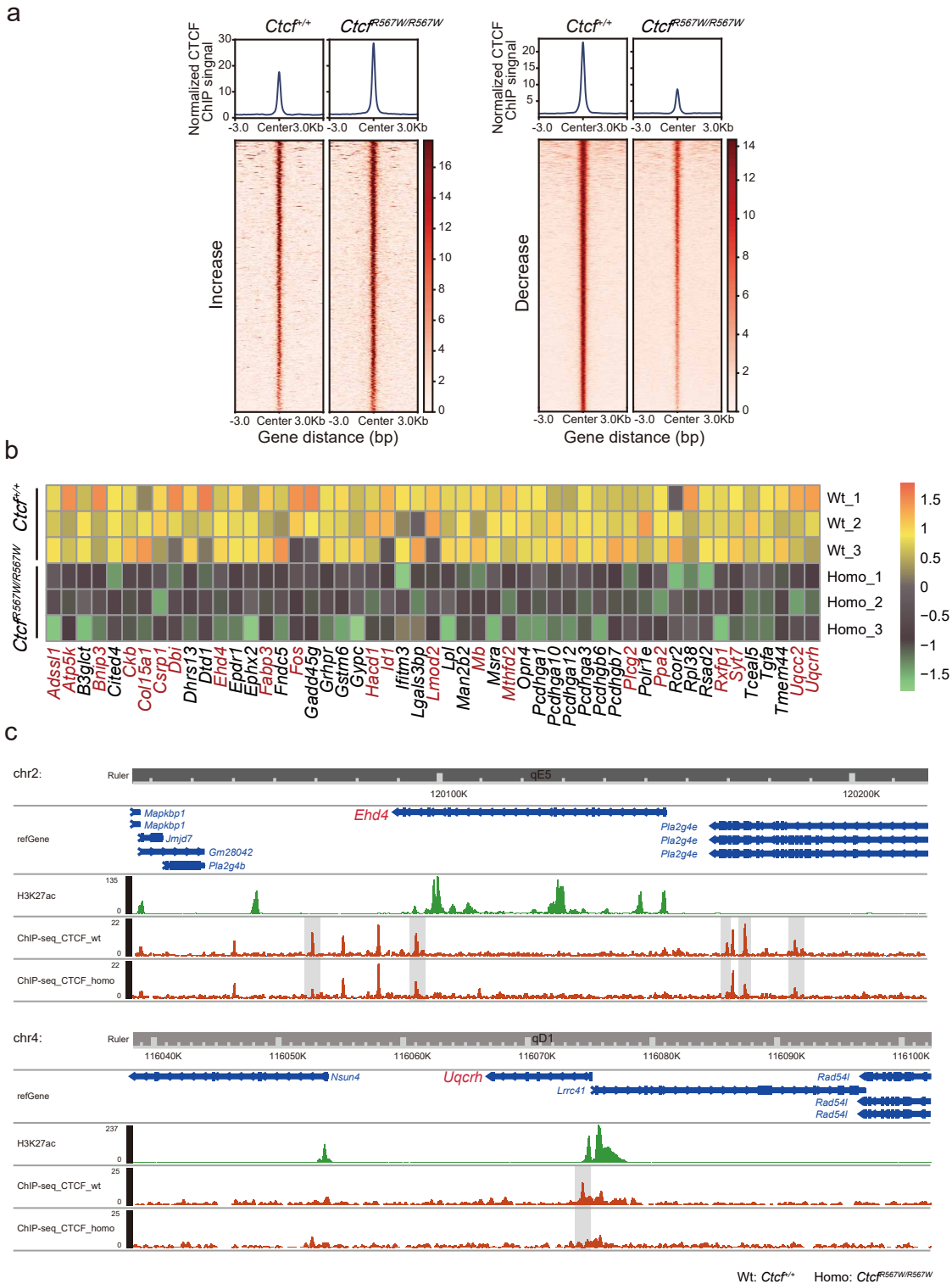

Supplement: Supplementary file 4 — Figure S4. Correlation analysis between changes in CTCF peaks and alterations in gene expression in the CTCF‐R567W mutation. (a) Heatmaps displaying CTCF ChIP‐seq signals at regions exhibiting differential binding sites in wild‐type versus Ctcf R567W/R567W mutation. Average profiles are shown above the corresponding heatmaps. (b) Heatmaps revealing 51 downregulated genes with decreased CTCF peaks nearby. Genes related to myocardial development are highlighted in red. (c), ChIP‐seq signal tracks providing insights into differential CTCF occupancy near Ehd4 and Uqcrh genes in wild‐type and Ctcf R567W/R567W mutation. [file CPR-58-e13783-s002.pdf]

Figure S5

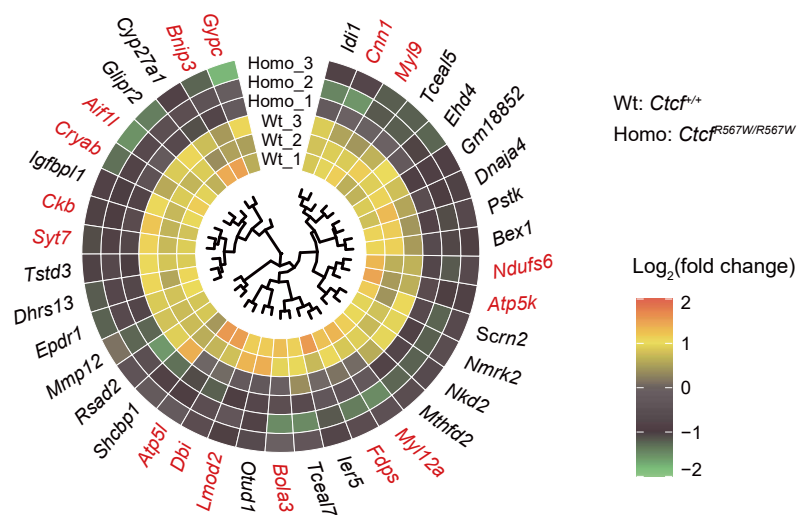

Supplement: Supplementary file 5 — Figure S5. Association analysis of RNA‐seq and Hi‐C data. Ring heatmap illustrating downregulated genes adjacent to altered TADs. Genes involved in myocardial development pathways are highlighted in red. [file CPR-58-e13783-s009.pdf]
